# The effect of jet lag on the human brain: A neuroimaging study

**DOI:** 10.1002/hbm.24945

**Published:** 2020-03-03

**Authors:** Feifei Zhang, Weikai Li, Huiru Li, Shaobing Gao, John A. Sweeney, Zhiyun Jia, Qiyong Gong

**Affiliations:** ^1^ Huaxi MR Research Center (HMRRC), Department of Radiology West China Hospital of Sichuan University Chengdu China; ^2^ College of Computer Science & Technology Nanjing University of Aeronautics & Astronautics (NUAA) Nanjing China; ^3^ College of Computer Science Sichuan University Chengdu China; ^4^ Department of Psychiatry and Behavioral Neuroscience University of Cincinnati Cincinnati Ohio; ^5^ Department of Nuclear Medicine, West China Hospital Sichuan University Chengdu China; ^6^ Psychoradiology Research Unit of Chinese Academy of Medical Sciences Sichuan University Chengdu China

**Keywords:** circadian rhythm disorder, fMRI, psychoradiology, graph theory, functional connectivity, jet lag

## Abstract

Jet lag is commonly experienced when travelers cross multiple time zones, leaving the wake–sleep cycle and intrinsic biological “clocks” out of synchrony with the current environment. The effect of jet lag on intrinsic cortical function remains unclear. Twenty‐two healthy individuals experiencing west‐to‐east jet lag flight were recruited. Brain structural and functional magnetic resonance studies, as well as psychological and neurohormonal tests, were carried out when participants returned from travel over six time zones and 50 days later when their jet lag symptoms had resolved. During jet lag, the functional brain network exhibited a small‐world topology that was shifted toward regularity. Alterations during jet lag relative to recovery included decreased basal ganglia‐thalamocortical network connections and increased functional connectivity between the medial temporal lobe subsystem and medial visual cortex. The lower melatonin and higher thyroid hormone levels during jet lag showed the same trend as brain activity in the right lingual gyrus. Although there was no significant difference between cortisol measurements during and after jet lag, cortisol levels were associated with temporal lobe activity in the jet lag condition. Brain and neuroendocrine changes during jet lag were related to jet lag symptoms. Further prospective studies are needed to explore the time course over which jet lag acts on the human brain.

## 1 INTRODUCTION

Jet lag is an exogenous circadian rhythm disorder following long‐distance air travel across multiple time zones (Herxheimer, 2008; Morgenthaler et al., 2007). The features of jet lag include fatigue, headache, irritability, poor concentration, gastrointestinal disorders, cognitive deficits, and sleep difficulties (Gander, Nguyen, Rosekind, & Connell, 1993; Herxheimer, 2014; Sack, 2010; Waterhouse, Reilly, & Atkinson, 1997). In athletes, jet lag can influence cognitive function (Drust, Waterhouse, Atkinson, Edwards, & Reilly, 2005), motivation (Reilly, Waterhouse, & Edwards, 2008), and muscle force (Reilly, Atkinson, & Budgett, 2001; Wright et al., 1983).

Structural magnetic resonance imaging (MRI) research (Cho, 2001) has reported temporal lobe atrophy and spatial cognitive deficits associated with chronic jet lag. Acute jet lag has been related to functional brain changes. One study of short‐term jet lag using resting‐state functional MRI showed decreased brain function in the anterior nodes of the default‐mode network (DMN) (Coutinho et al., 2015). Previous studies have been limited by case–control rather than longitudinal designs and relatively small sample sizes. There is still a lack of systematic neuroimaging research on the effects of jet lag in humans.

Changes in circadian neuroendocrine systems that become out of synchrony with environmental day/night cycles are believed to be a central cause of jet lag and include melatonin, cortisol, and thyroid‐stimulating hormone (TSH; Morgenthaler et al., 2007; Zisapel, 2018). The production cycle of melatonin keeps pace with the sleep–wake cycle, rising during the evening before sleep (Herxheimer & Petrie, 2002) and peaking at approximately 2:00 a.m. (Carter & Juurlink, 2012). Under the guidelines of the American Academy of Sleep Medicine, melatonin has been recommended to reduce jet lag symptoms (Morgenthaler et al., 2007). Studies have shown robust relations between the neuroendocrine system and activity in functional brain systems. Subcortical (midbrain, cerebellum, basal ganglia, and thalamus) and neocortical regions have been shown to be regulated by circadian rhythms and melatonin (Muto et al., 2012). TSH and cortisol were also found to be regulated both by circadian rhythm and sleep (Hirschfeld et al., 1996). A previous study reported a negative relationship between cortisol levels and volume of the right temporal lobe (Cho, 2001).

However, alterations in functional brain systems related to jet lag and its neuroendocrine associations remain to be fully clarified. MRI studies of individuals during jet lag compared to when entrained to the local daylight cycle can address this issue. The primary objective of this study was to detect whole‐brain functional changes after jet lag. We evaluated a team of neuroimaging researchers after their return from the 6‐day annual meeting of the International Society for Magnetic Resonance in Medicine (ISMRM) in Honolulu, Hawaii, United States to Chengdu, China (which crosses six time zones), and we followed them after their jet lag symptoms had been resolved. Behavioral and neuroendocrine studies were performed in parallel with neuroimaging to investigate the mechanisms of brain and behavioral changes. The goal of the present study was to compare the whole‐brain gray matter network connections and network properties in subjects during jet lag with recovery. We predicted lower global efficiency metrics when participants experienced jetlag. The second objective was to detect changes in the DMN and in jet lag‐related hormone levels. In light of previous studies, we expected to find lower functional connectivity in DMN, higher cortisol levels, and lower melatonin level during jet lag. The third objective was to explore the relationship between alterations of brain function and jet lag‐related symptoms, predicting that greater brain alterations would be associated with greater jet lag symptoms.

## 2 MATERIALS AND METHODS

### 2.1 Participants

**Figure 1:**
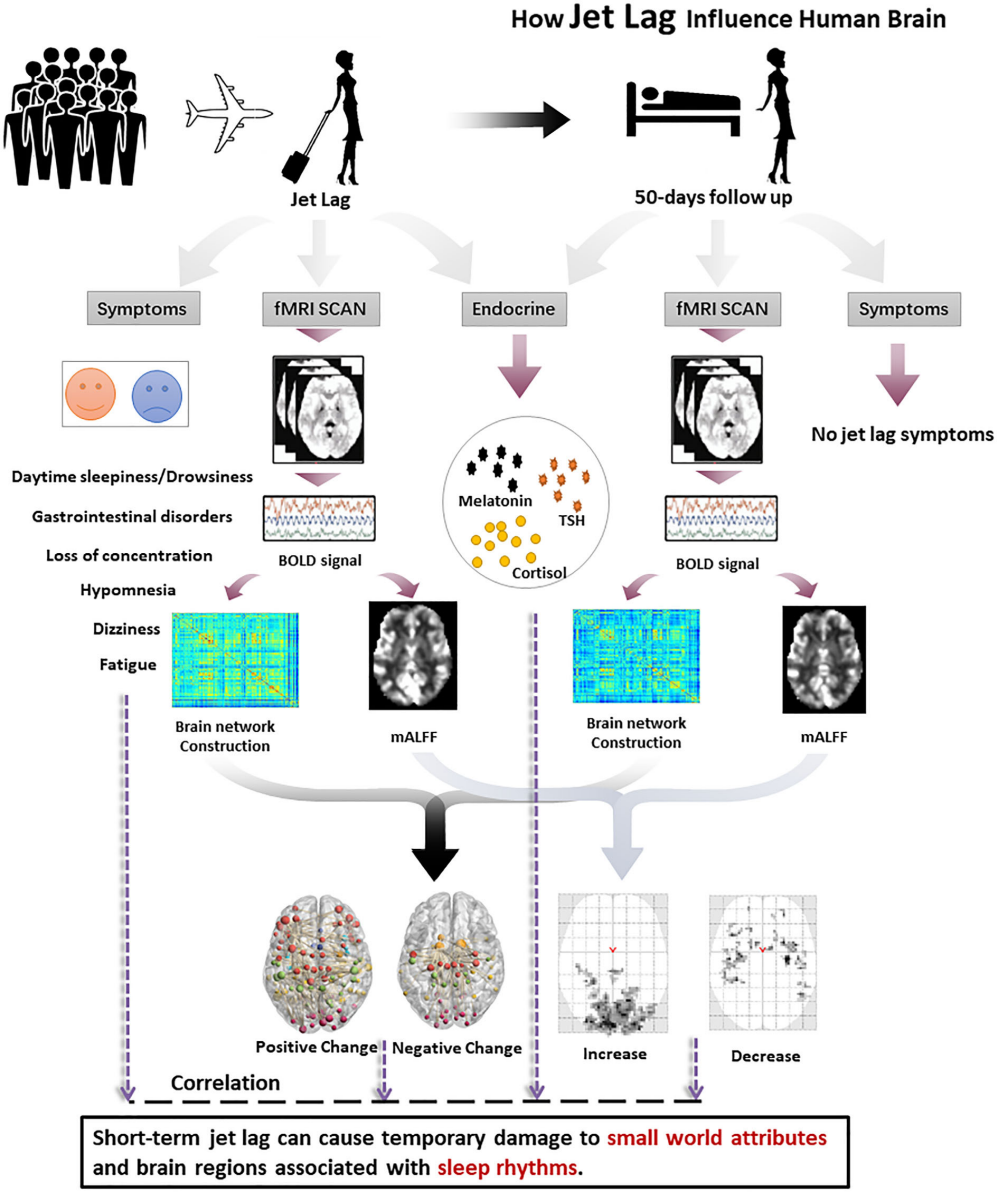
The flow chart showed that 22 subjects who had traveled on a long‐haul flight across six time zones were tested when they returned to China and again 50 days later. Whole‐brain BOLD signals were extracted, and brain networks were constructed to analyze alterations in functional connections

This study was approved by the West China Hospital Ethics Committee of Sichuan University. Written informed consent was obtained from all participants who were recruited and studied from April 2017 to June 2017 following their direct return to Chengdu, China from a 6‐day stay at the 2017 annual meeting of the ISMRM in Hawaii (Figure 1). This route covered 107° longitude and 10° latitude, spanning six time zones, taking 16 hr and passing approximately 30% of the way around the globe. Study exclusion criteria were as follows: (a) taking drugs to sleep or reduce jet lag symptoms; (b) taking other flights in the month prior to testing; (c) somatic or psychiatric disorders, neurological disorders, contraindications for MRI scans, including pregnancy; (d) left‐handedness; (e) age less than 18 or over 60 years; and (f) history of current or ongoing serious medical problems. Twenty‐two Han Chinese ethnicity and right‐handed participants (11 males and 11 females with a mean age of 28.9 ± 5.6 years, ranging from 23 to 46 years) traveled together and were included in the statistical analyses (Table 1). One subject was excluded because of other cross‐time zone flights during the follow‐up period. Another subject was excluded for excessive head motion during the scan.

**Table 1:** Demographic data, behavioral, and neuroendocrine characteristics of study participants

Characteristic	Jet lag	50‐day follow‐up	*p* value
Sample size	22		NA
Age (years)	28.59 ± 5.66		NA
Gender (M/F)	10/12		NA
Handedness (R/L)	22/0		NA
Positive emotion	17.77 ± 6.11	25.59 ± 8.04	<.001^a^
Negative emotion	14.27 ± 4.72	13.27 ± 3.70	.233
Anxiety sore	42.64 ± 11.25	35.95 ± 9.77	.020^a^
TSH (mU/L)	4.64 ± 2.21	2.83 ± 2.07	.003^a^
COR (nmol/L)	399.80 ± 159.96	395.50 ± 129.74	.899
MEL (ng/ml)	1.37 ± 0.78	1.83 ± 0.99	.049

*Note*: Data are presented as the mean ± SD. The *p* value was obtained using a paired *t* test.

Abbreviations: TSH, thyroid‐stimulating hormone; COR, cortisol; MEL, melatonin; JLD, jet lag disorder.

a Region considered significant after multiple‐comparison correction.

Brain structural and functional MRI and blood draw for neurohormone assays, including melatonin, cortisol, and TSH, were performed the day after the participants returned to China (between 6 a.m. and 8 a.m. Hawaii time; Table 1). First, all participants were assessed with the Positive and Negative Affect Schedule (Crawford & Henry, 2004), the State Anxiety Inventory (Ramanaiah, Franzen, & Schill, 1983), the Columbian Jet Lag Scale (Spitzer et al., 1999), and the Epworth Sleepiness Scale (Bollettini et al., 2017). The Columbian Jet Lag Scale is used to assess the presence and severity of jet lag symptoms (Spitzer et al., 1999). Then, all participants were asked to sit and rest for more than 5 min before hormone testing. Finally, MR scanning was performed. Participants were re‐examined approximately 50 days after the first test, by which time their jet lag symptoms had resolved. The second time point for testing was at the same relative time in the solar cycle (in Chengdu time) as the first time.

### 2.2 MRI data acquisition

Blood oxygen level‐dependent‐based fMRI and 3D T1‐weighted structural MRI scans were obtained using a Siemens 3.0 Tesla MR system (Tim Trio; Siemens Healthineers, Erlangen, Germany). Participants were asked to close their eyes and try not to think about anything during the scan. The lights were dimmed, and the participants' heads were supported by foam blocks to reduce head movement. The fMRI data were obtained using an echo‐planar imaging sequence: repetition time/echo time (TR/TE) = 2,000/30 ms; flip angle = 90°; slice thickness = 3.8 mm; matrix = 64 × 64; field of view (FOV) = 240 × 240 mm^2^, single‐voxel size = 3.75 × 3.75 × 3.8 mm^3^. The scanning time lasted 410 s (205 volumes). Three‐dimensional T1‐weighted images were obtained with the following parameters: TR/TE = 1,900/2.26 ms, flip angle = 9°, 175 axial slices with slice thickness = 1 mm, FOV = 256 × 256 cm^2^, single‐voxel size = 1 × 1 × 1 mm^3^.

### 2.3 Image preprocessing

All brain images were collected parallel to the anterior and posterior commissure line. Image preprocessing was performed in SPM12 (http://www.fil.ion.ucl.ac.uk/spm) using Data Processing Assistant for Resting‐State fMRI (DPARSF, http://rfmri.org/DPARSF; Chao‐Gan & Yu‐Feng, 2010). The process included removing the first 10 time points and then implementing slice‐timing and head‐movement corrections. Head motion was required to be less than 2.5 mm and 2.5° in any direction. Then, all data were regressed with 24‐parameter motion correction, and white matter and CSF signals were removed. The functional images were registered to each individual's 3D T1 structural images. Images were normalized into Montreal Neurological Institute (MNI) space, and 3‐mm isotropic voxels were resampled in the same step. Finally, smoothing (full‐width at half‐maximum: 6 mm), linear detrending and bandpass filtering (0.01–0.10 Hz) were conducted to remove the effects of low‐frequency drift and high‐frequency physiologic noise. To reduce the effects of head movement, we implemented a scrubbing procedure using a method that deleted images with larger head movements, defined as frame‐wise displacement (FD) > 0.5 mm (Power, Barnes, Snyder, Schlaggar, & Petersen, 2012), and participants with more than 20% of their volumes deleted were excluded (mean FD after srubbing was 0.154 ± 0.102 mm) (see Supporting Information for more information).

### 2.4 Brain network construction and parameter calculation

Brain networks for each participant were constructed in Gretna (http://www.nitrc.org/projects/gretna/) using a template containing 246 brain regions (Fan et al., 2016). When calculating network properties, we selected a sparsity range from 0.03 to 0.44 (described in detail elsewhere, Zhang et al., 2011) with a step size of 0.01 to establish a binary network. The global and nodal properties of each network at different levels of sparsity were calculated, after which the area under the curve formed across the sparsity range was determined. The four global properties examined included the following: (a) small‐world parameters (for definitions, see Watts & Strogatz, 1998), including the clustering coefficient, characteristic path length, normalized clustering coefficient, normalized characteristic path length, and small‐worldness; (b) local and global efficiency (Latora & Marchiori, 2001); (c) hierarchy, which reflects the possible existence of smaller modules embedded in larger modules (Rubinov & Sporns, 2010); and (d) assortativity, the correlation coefficient between the degrees of all the two relative ends on a link. According to a previous report, networks with positive correlation coefficients may have relatively flexible scores for highly interconnected hubs (Rubinov & Sporns, 2010). The nodal degree, centrality, and betweenness were calculated as nodal properties.

Furthermore, the mean amplitude of low‐frequency fluctuation (mALFF) was obtained using the unscrubbed data (mean FD before srubbing is 0.176 ± 0.161 mm). Scrubbed data were used for network modeling because removal of head motion artifact is important for modeling network features, while for ALFF the raw continuous data distribution without gap needs to be examined. For this analysis, the filtered time series from each voxel was converted into the frequency domain by a fast Fourier transform to obtain a power spectrum. Since the power of a given frequency is proportional to the square of the amplitude of the frequency component, the square root at each frequency of the power spectrum was calculated, and then a root means square of activity between 0.01 and 0.10 Hz was obtained at each voxel. The square root of this average is considered the ALFF value for a voxel. Individual ALFF maps were also divided into the mALFF values from the whole‐brain mask to standardize the data across participants and time.

### 2.5 Statistical analysis

Paired *t* tests were performed to compare data collected during the jet lag period and after recovery for the psychological questionnaires, hormone data, and area under the curve of the topological properties. To identify altered functional connectivity, we use a paired *t* test to compare individual network connections after Fisher's *r*‐to‐*z* transformation, which was used to improve normality for these connectivity estimates. Cluster voxels with differences in the correlations of mALFF values with other brain regions were identified. All statistical analyses were corrected using the false discovery rate procedure (FDR = 0.05). Finally, Pearson correlation analysis was used to explore correlations between brain parameters and psychological and hormone data.

### 2.6 Data availability

Data supporting the results of this study can be obtained from the corresponding authors upon reasonable request.

### 2.7 Code availability

All the code for this study will be available through the corresponding authors.

## 3 RESULTS

### 3.1 Demographic and clinical characteristics

Analysis of behavioral questionnaires showed that after the flight to China at the first testing point, most participants reported excessive daytime sleepiness (12/22). Some complained of fatigue (9/22), reduced concentration (7/22), gastrointestinal problems (3/22), hypomnesia (2/22), and dizziness (1/22). All individuals reported varying degrees of drowsiness (a mean score of 9.0 ± 2.0), and five individuals scored more than 11 points on the Epworth sleepiness scale. Positive mood during the jet lag test was significantly lower (*p* < .001, FDR‐corrected) and anxiety was significantly higher (*p* = .020, FDR‐corrected) than after recovery from jet lag.

A similar analysis was performed with hormone levels during and after recovery from jet lag. Relative to assessments after recovery from jet lag, TSH values were significantly higher (*p* = .003, FDR‐corrected) while melatonin values were lower (*p* = .049, uncorrected) during jet lag. No significant differences were observed in cortisol values (*p* = .899).

### 3.2 Global topological organization of brain function during jet lag

**Figure 2:**
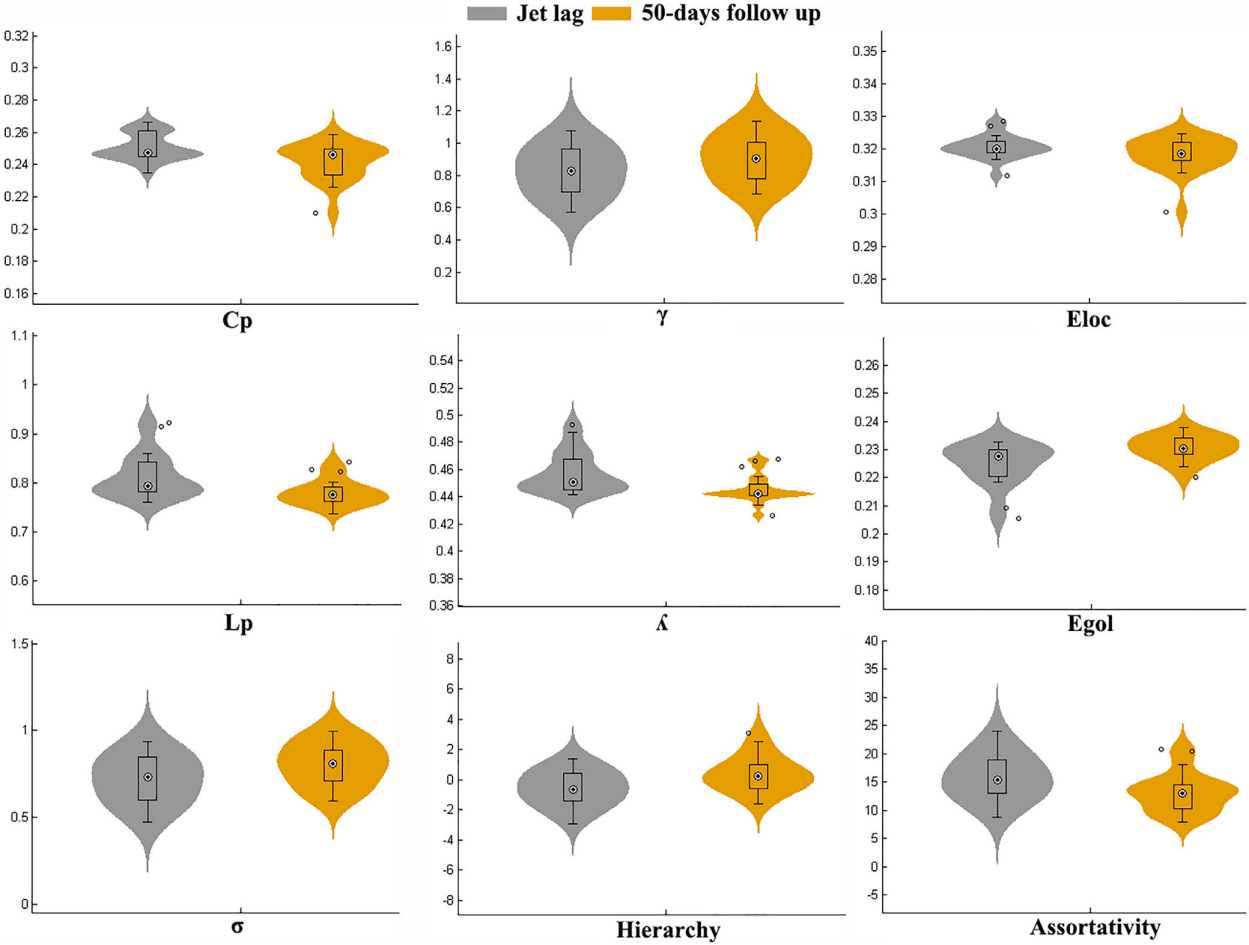
Violin plots show differences in topologic properties of brain functional connectivity. During jet lag, participants showed a significantly higher clustering coefficient (Cp, *p* = .002), characteristic path length (Lp, *p* = .0005), normalized characteristic path length (*λ*, *p* = .0003) and assortativity (*p* = .0007) and a lower normalized clustering coefficient (γ, *p* = .007), small‐worldness (σ, *p* = .001), globe efficiency (Egol, *p* = .003) and hierarchy (*p* = .001)

Compared with recovery, participants while experiencing jet lag showed a small‐world topology, but there was a shift toward greater network regularity during jet lag in terms of the global properties, including the clustering coefficient (*t* = 3.51, *p* = .002), characteristic path length (*t* = 4.04, *p* = .0005), normalized characteristic path length (*t* = 4.23, *p* = .0003), and assortativity (*t* = 3.93, *p* = .0007). We also observed reduced normalized clustering coefficient (*t* = −2.98, *p* = .007), small‐worldness (*t* = −3.67, *p* = .001), global efficiency (*t* = −4.24, *p* = .003), and hierarchy (*t* = −3.76, *p* = .001) (Figure 2). All results were significant after correction using the FDR method.

### 3.3 Local topological organization of brain function during jet lag

Compared with recovery, nodal efficiency was lower during jet lag in the orbital frontal gyrus, left precuneus, left cingulate gyrus, bilateral basal ganglia (including bilateral ventral caudate, right dorsal caudate, bilateral nucleus accumbens, and left ventromedial putamen), and bilateral thalamus (*p* < .05, FDR‐corrected). Nodal centrality was decreased in the left ventral caudate, right nucleus accumbens, right medial frontal thalamus, right rostral temporal thalamus, and bilateral lateral frontal thalamus (*p* < .05, FDR‐corrected). No regions showed increased nodal efficiency or centrality during jet lag (Table 2).

**Table 2:** Regions showing decreased nodal centrality during jet lag

Label	Brain regions	Nodal efficiency			Nodal degree			Nodal betweenness		
		Jet lag	50‐day follow‐up	*p* value	Jet lag	50‐day follow‐up	*p* value	Jet lag	50‐day follow‐up	*p* value
41	OrG_L_6_1	0.221 ± 0.029	0.240 ± 0.022	.001^a^	21.294 ± 9.233	26.596 ± 7.956	.010	61.393 ± 44.700	63.014 ± 38.710	.889
42	OrG_R_6_1	0.219 ± 0.026	0.241 ± 0.026	<.001^a^	20.246 ± 8.174	27.275 ± 9.230	.002	47.185 ± 27.747	76.144 ± 46.505	.009
154	PCun_R_4_4	0.218 ± 0.027	0.235 ± 0.022	.002^a^	19.813 ± 7.91	24.151 ± 8.164	.018	58.646 ± 67.12	60.704 ± 37.520	.887
175	CG_L_7_1	0.212 ± 0.030	0.231 ± 0.021	.003^a^	18.296 ± 9.245	22.926 ± 7.563	.029	46.020 ± 28.185	44.113 ± 25.309	.790
187	CG_L_7_7	0.216 ± 0.027	0.234 ± 0.021	.003^a^	18.891 ± 8.340	23.813 ± 7.682	.020	58.665 ± 49.251	57.649 ± 26.061	.930
219	BG_L_6_1	0.162 ± 0.039	0.196 ± 0.029	<.001^a^	7.195 ± 5.80	12.246 ± 7.052	<.001^a^	24.950 ± 26.354	36.097 ± 35.914	.210
220	BG_R_6_1	0.187 ± 0.039	0.214 ± 0.028	<.001^a^	12.564 ± 8.063	17.471 ± 8.190	.007	62.414 ± 30.630	69.332 ± 54.013	.627
223	BG_L_6_3	0.229 ± 0.032	0.254 ± 0.024	<.001^a^	24.543 ± 10.434	32.036 ± 9.236	.003	139.697 ± 116.065	147.999 ± 89.882	.815
224	BG_R_6_3	0.216 ± 0.033	0.244 ± 0.023	<.001^a^	20.046 ± 10.071	28.159 ± 8.598	<.001^a^	83.659 ± 61.547	104.561 ± 81.922	.328
225	BG_L_6_4	0.204 ± 0.039	0.228 ± 0.023	.001^a^	17.308 ± 9.202	22.721 ± 7.285	.008	61.489 ± 46.082	45.750 ± 47.584	.143
228	BG_R_6_5	0.139 ± 0.037	0.163 ± 0.035	.001^a^	4.530 ± 2.708	6.372 ± 4.57	.046	14.812 ± 14.994	21.379 ± 30.991	.224
231	Tha_L_8_1	0.194 ± 0.042	0.225 ± 0.037	<.001^a^	15.663 ± 8.437	22.712 ± 11.130	.005	106.175 ± 89.236	92.129 ± 58.135	.447
232	Tha_R_8_1	0.197 ± 0.042	0.229 ± 0.033	<.001^a^	15.663 ± 8.494	24.240 ± 11.4832	<.001^a^	83.633 ± 45.792	101.844 ± 49.082	.140
233	Tha_L_8_2	0.208 ± 0.042	0.232 ± 0.033	.002^a^	19.700 ± 9.935	24.798 ± 11.189	.017	79.31 ± 64.403	62.086 ± 47.976	.263
234	Tha_R_8_2	0.213 ± 0.046	0.239 ± 0.037	.001^a^	21.167 ± 11.775	27.526 ± 11.995	.004	76.790 ± 57.133	78.097 ± 68.352	.928
237	Tha_L_8_4	0.162 ± 0.037	0.192 ± 0.035	<.001^a^	8.193 ± 5.219	12.663 ± 8.151	.001	46.178 ± 64.288	38.057 ± 40.461	.545
238	Tha_R_8_4	0.173 ± 0.037	0.209 ± 0.036	<.001^a^	10.393 ± 6.729	17.621 ± 9.844	<.001^a^	43.934 ± 38.764	62.7870 ± 36.994	.142
243	Tha_L_8_7	0.148 ± 0.041	0.174 ± 0.044	.002^a^	6.321 ± 5.910	9.584 ± 7.329	.006	17.438 ± 26.929	16.267 ± 19.150	.866
245	Tha_L_8_8	0.215 ± 0.046	0.246 ± 0.039	<.001^a^	22.069 ± 12.217	30.459 ± 13.014	<.001^a^	107.774 ± 82.645	127.857 ± 100.285	.369
246	Tha_R_8_8	0.211 ± 0.045	0.244 ± 0.042	<.001^a^	20.905 ± 11.548	30.069 ± 13.429	.001^a^	111.001 ± 83.631	116.026 ± 64.589	.802

*Note*: Data are means ± standard deviations unless otherwise indicated. Regions were considered to have regional changes in the participants during jet lag if they exhibited significant between jet lag and recovery difference (*p* < .05, false discovery rate corrected) in at least one of the three nodal centralities.

Abbreviations: OrG, orbital gyrus; Pcun, precuneus; CG, cingulate gyrus; BG, basal ganglia; Tha, thalamus.

a Corrected for multiple comparisons using FDR procedure.

### 3.4 Global network functional connections in jet lag

**Figure 3:**
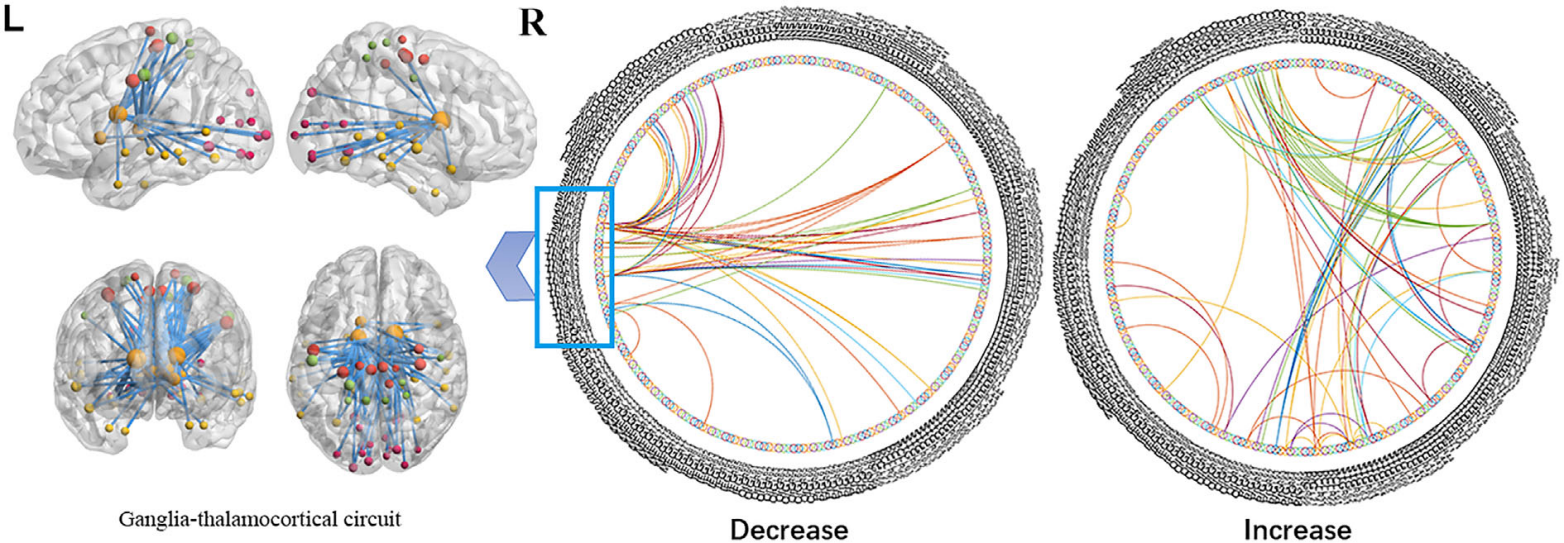
A paired *t* test was conducted across the whole brain, including 246 nodes, in the jet lag condition. The inner connections of frontotemporal cortex were enhanced. The relationship between the visual loop and other cortical regions (temporal lobe, parietal lobe, frontal lobe) were enhanced. The basal ganglia‐thalamocortical circuit was decreased

Comparison of data obtained during recovery and jet lag revealed less robust connections within the following regions during jet lag compared to recovery: precentral gyrus, paracentral gyrus, temporal gyrus, postcentral gyrus, occipital gyrus, and subcortical nuclei. Almost all of the lower functional connections involved associations with subcortical nuclei, especially the bilateral caudate and thalamus. Some connections of the frontotemporal cortex were strengthened. Additionally, some enhancement of connections between the occipital cortex and temporal, parietal, and frontal cortices was observed during jet lag compared with recovery (Figure 3).

### 3.5 Brain functional changes

Participants had lower ALFF values in the right angular gyrus, right caudate, right hippocampus extending to the parahippocampus, and bilateral inferior temporal gyrus and increased ALFF in bilateral lingual gyrus (*p* < .001, FDR‐corrected) during jet lag compared with recovery (Table 3).

**Table 3:** Brain regions in which ALFF values were different during jet lag and after recovery in a whole‐brain analysis

Location	MNI			Cluster size mm^3^	*p* value
	*x*	*y*	*z*		
*Increases*					
Lingual_L	−12	−84	−9	97	<.001^a^
Lingual_R	18	−69	−12	82	<.001^a^
Thalamus_L	−12	−21	−3	11	.001
*Decreases*					
Temporal_Inf_L	−48	−9	−27	143	<.001^a^
Temporal_Inf_R	42	3	−45	92	<.001^a^
Hippocampus_R	30	−15	−18	42	<.001^a^
Caudate_R	18	12	24	114	<.001^a^
Caudate_L	−6	6	−3	5	.001
Angular_R	57	−54	36	41	<.001^a^

a Corrected for multiple comparisons using FDR procedure.

### 3.6 Relationship between global brain functional parameters and behavioral assessments

Only two relationships survived multiple‐comparison correction with FDR: (a) the nodal centrality of the left inferior frontal gyrus was correlated with positive emotion scores (*p* = .0001, FDR‐corrected; *r* = 0.71) during jet lag. (b) The lower functional activity in the right hippocampal gyrus relative to retest after recovery was correlated with increased anxiety during jet lag (*p* = .034, FDR‐corrected, *r* = −0.61).

**Figure 4:**
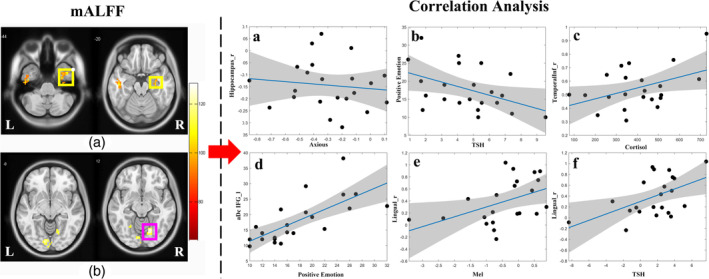
The functional activity was significantly lower in the bilateral inferior temporal gyrus (a, left) and right hippocampus (a, right) and increased in the bilateral lingual gyrus (b). Two correlations survived after multiple‐comparison correction: The functional activity alterations in the right hippocampal gyrus were negatively correlated with anxiety scores (*p* = .034, false discovery rate corrected, a). The nodal centrality of the left inferior frontal gyrus was positively correlated with positive emotional score during jet lag (*p* = .0001, false discovery rate corrected, d). Other significant relationships that were nominally significant but did not meet the threshold of multiple‐comparison correction included: A negative correlation between TSH levels and positive emotion scores during jet lag (*p* = .039, b). Right inferior temporal gyrus activity (*p* = .044, c) was positively correlated with cortisol levels during jet lag. Positive correlations were found between the brain activity of right lingual gyrus and melatonin (*p* = .031, e) and TSH (*p* = .028, f)

Other nominally significant correlations that did not survive FDR correction include: negative correlations between TSH and positive emotion scores during jet lag (*p* = .039, uncorrected, *r* = −0.44) and between melatonin and drowsiness (*p* = .026, uncorrected, *r* = −0.47). Increased TSH (*p* = .028, uncorrected, *r* = 0.47) and decreased melatonin (*p* = .031, uncorrected, *r* = −0.46) were related to increased activity in the right lingual gyrus. Right inferior temporal gyrus activity during jet lag was positively correlated with cortisol (*p* = .044, uncorrected, *r* = 0.43). Scores on the Columbian Jet Lag Scale were negatively related to left inferior temporal gyrus activity (*p* = .026, uncorrected, *r* = −0.47) and melatonin levels (*p* = .021, uncorrected, *r* = −0.49; Figure 4).

## 4 DISCUSSION

The neuroimaging results demonstrate that the whole‐brain functional network exhibited a trend toward regularization during jet lag compared with recovery. Nodal efficiency and centrality were lower mainly in the basal ganglia and thalamus, and some of these regions had functional activity alterations based on the mALFF analyses. In addition, correlational analyses demonstrated a relationship between jet lag symptoms and neuroimaging findings. Additionally, brain changes and neuroendocrine changes during jet lag showed the same trend as jet lag symptoms.

The brain networks of participants during jet lag exhibited small‐worldness but trended toward regularization. In terms of the global properties, the clustering coefficient, characteristic path length, and normalized characteristic path length were significantly higher, whereas the normalized clustering coefficient, small‐worldness, and global efficiency were lower. The human brain has the property of small‐worldness, an optimal balance between separation and integration of information processing (Sporns, Tononi, & Kotter, 2005). During jet lag, the efficiency of the whole brain decreased and the properties of the whole‐brain network changed. We also found that the assortativity showed an increase during the experience of jet lag. According to a previous review, the assortativity and nodal degree are indirect quantitative indicators of resilience reflecting network vulnerability (Rubinov & Sporns, 2010). All in all, the present study found that the nodal degree, hierarchy, and network efficiency of the functional network were all lower but the assortativity was higher during jet lag. These findings suggest that when the efficiency of the functional network was decreased, priority was given to ensuring the connections of the hub‐like nodes for maintaining brain function.

During jet lag, the nodal centrality and efficiency were altered, especially in the basal ganglia and thalamus. The functional activity and the connection of caudate nucleus bilaterally with the cerebral cortex were lower during jet lag. Additionally, during jet lag, relatively higher activity was found in the thalamus, but lower connections were found between thalamus and cerebral cortex, including the superior parietal gyrus, the postcentral, precentral and paracentral gyri, the precuneus, the bilateral medioventral and lateral occipital cortex, the superior temporal gyrus, and the parahippocampal gyrus. A total sleep deprivation (TSD) study reported decreased thalamocortical functional connectivity in fMRI (Shao et al., 2013).

The thalamus is a key region involved in maintaining alertness and arousal, and plays an important role in integrating information from widespread regions of the neocortex (Postuma & Dagher, 2006). Previous findings have suggested that TSD can impair thalamic integration, which was associated with decreased attention, daytime sleepiness, and fatigue (Plante et al., 2014; Postuma & Dagher, 2006), which is consistent with our findings regarding jet lag symptoms. Previous task‐based MRI studies of sleep deprivation revealed decreased thalamic activity (Chee et al., 2006; Chee & Choo, 2004). The difference may be because prior research incorporated tasks during scanning, and activity in thalamus may be changed because its role in supporting performance on the particular task employed (Plante et al., 2014). In this context, over‐activation of the thalamus in the resting state may reflect an effort to maintain alertness to compensate for the disconnection of the thalamic cortical network, a process previously referred to as compensatory adaptation (Chee & Choo, 2004).

Activity in the inferior parietal gyrus, which plays a crucial role in sustained attention (Niu et al., 2018), was reduced during jet lag. The same results were previously reported during their task‐based studies (Chee et al., 2006; Chee & Choo, 2004) and were confirmed by a positron emission tomography study (Thomas et al., 2000). Decreased activation of the parietal cortex has been associated with decreased performance in tasks, such as serial subtraction (Thomas et al., 2000) and working memory tasks (Chee et al., 2006), which may contribute to jet lag symptoms such as impairments in memory and attention. In the present study, the connections among the superior parietal gyrus, postcentral gyrus, thalamus, precentral gyrus, and paracentral gyrus were weakened during jet lag, suggesting that information networks dependent on the integrative function of thalamus in thalamocortical systems and information processing may be effectively disrupted.

Activity in thalamus, basal ganglia, and occipital lobe is regulated by circadian rhythms (Muto et al., 2012). The current study found higher occipital cortical activity, similar to previous studies (Zhu et al., 2016). In addition, higher connectivity was found between the occipital lobe and middle frontal cortex, temporal lobe (inferior temporal gyrus, fusiform gyrus, and parahippocampus), and parietal lobe. The function of the occipital cortex is controlled by top‐down regulation mechanisms as well as by thalamocortical drive (Karten, Pantazatos, Khalil, Zhang, & Hirsch, 2013). Increased functional connections between the medial temporal lobe subsystem (Andrews‐Hanna, Reidler, Sepulcre, Poulin, & Buckner, 2010) and occipital regions may contribute to hyperactivity of bilateral occipital gyrus in jet lag. Higher activation in occipital cortex during jet lag may be related to alterations in circadian rhythms (Muto et al., 2012), especially in medial occipital cortex (Gorfine & Zisapel, 2009; Muto et al., 2016). According to a previous study, the occipital lobe not only plays a role in visual information processing but also performs functions related to visual cognition (Zhao et al., 2016). Although the current results are contrary to our expectations in cognitive‐related brain regions and network weakening, they reflect another effect of jet lag on the brain. These results need to be further explored.

The Pearson correlation analysis in the current study revealed an association between endocrine hormones and imaging findings. The Pearson correlation analysis found that melatonin was negatively related to drowsiness and jet lag scores and that melatonin levels were lower during jet lag. However, the relationship between jet lag symptoms and melatonin did not survive correction for multiple corrections.

Melatonin levels are reduced at night during jet lag, a finding that is consistent with the significant benefit of oral melatonin in relieving jet lag symptoms (Herxheimer & Petrie, 2002; Iggena, Winter, & Steiner, 2017; Morgenthaler et al., 2007). In the present study, we observed a trend for a relationship between decreases in melatonin and increases in right occipital lobe activity during jet lag. In typical sleep patterns in healthy individuals, increased melatonin in the afternoon was found to reduce activation of the occipital cortex even when melatonin levels were below a threshold level (Gorfine & Zisapel, 2009). We observed a nominally significant trend for a relationship between the two parameters in our study. Further studies are needed to explore this correlation.

In the present study, TSH levels were significantly higher in the jet lag condition. TSH is regulated by both circadian rhythms and sleep (Hirschfeld et al., 1996). Jet lag can induce a prolonged elevation of TSH, which may be associated with the jet lag syndrome (Hirschfeld et al., 1996). However, there were no significant findings on the association of TSH and jet lag syndrome in the present study. However, increased TSH levels were associated with increased activation of the right occipital gyrus during jet lag. A longitudinal study found decreases in visuospatial (Wahlin, Bunce, & Wahlin, 2005) and memory (Hogervorst, Huppert, Matthews, & Brayne, 2008) abilities were accompanied by decreases in TSH levels. TSH and positive emotion scores showed opposite changes during jet lag relative to recovery. In our study, a positive correlation was found between decreased efficiency of the left inferior frontal gyrus and lower levels of positive emotions. This finding may be related to a disruption of inferior frontal gyrus which is well known to play an important role in emotional reactions and mood.

Another jet lag‐related hormone, cortisol, was found to be positively associated with the right inferior temporal gyrus function. According to a previous chronic jet lag study, cortisol is disruptive to hippocampus‐dependent cognition (Cho, 2001). In the jet lag condition, cortisol levels were relatively high, the comparison of levels between jet lag and recovery states were not significant, nor were relations between cortisol and brain function. The temporal lobe is thought to be related to cognition and memory. Although this negative correlation was not significant after correction, it indicates the contribution of cortisol to jet lag symptoms. The results are also consistent with previous studies (Cho, 2001). In conclusion, cortisol showed an adverse effect on the temporal gyrus and may also influence cognition. Notably, this correlation is very weak, and so the correlation analysis here should be considered exploratory.

In the present study, the activation of the right hippocampus was found to be lower during jet lag and positively correlated with anxiety. Task‐based fMRI studies have found that hippocampal–cortical interactions occur during memory and recall, and the amygdala regulates sleep‐related negative emotional changes in a way that affects hippocampal–neocortical dialog (Sterpenich et al., 2007). Sleep‐related positive emotions have been shown to be related to an enhancement of these hippocampal–cortical interactions (Sterpenich et al., 2007) in a study that also found decreased right hippocampal activation after TSD (Sterpenich et al., 2007). The previous study confirmed that the hippocampus affects sleep‐related emotions, and this result had also emerged in the current study.

## 5 LIMITATIONS

There are several factors that could have affected our results in the current study. First, the relatively small sample size in the current study may limit the generalizability of the results. Secondly, the present study lacks data before jet lag, comparing jet lag data to recovery not pre‐travel scans. Further studies will include initial baseline data from subjects without jet lag to increase the rigor of the results. Finally, the present study considered only the influence of short‐term jet lag. The difference between short‐term and chronic jet lag is still unclear, and further studies in this area are needed.

## 6 CONCLUSION

In summary, jet lag is a complex problem. This study explored the effect of jet lag from the perspective of physiology, neuroimaging, and behavioral science. The current results showed that short‐term jet lag can cause temporary jet lag symptoms, as evidenced by all subjects having no appreciable symptoms at the 50‐day follow‐up. In addition, our MRI results indicated that jet lag leads to a disruption of small‐world attributes and brain regions associated with sleep rhythms. We also found evidence that jet lag‐related hormone levels were associated with activity in specific brain regions.

## CONFLICT OF INTEREST

The authors declare no potential conflict of interest.

## Supporting information


**Supplementary Figure S1** The figure below shows mean FD across the entire time series for each subject. The blue line represents FD values before scrubbing, while the red line means the FD values after scrubbing. FD, frame‐wise displacement.

## Data Availability

Data availability Data supporting the results of this study can be obtained from the corresponding authors upon reasonable request. Code availability All the code for this study will be available through the corresponding authors.

## References

[hbm24945-bib-0001] Andrews‐Hanna, J. R. , Reidler, J. S. , Sepulcre, J. , Poulin, R. , & Buckner, R. L. (2010). Functional‐anatomic fractionation of the Brain's default network. Neuron, 65(4), 550–562. 10.1016/j.neuron.2010.02.005 20188659 PMC2848443

[hbm24945-bib-0002] Bollettini, I. , Melloni, E. M. , Aggio, V. , Poletti, S. , Lorenzi, C. , Pirovano, A. , … Benedetti, F. (2017). Clock genes associate with white matter integrity in depressed bipolar patients. Chronobiology International, 34(2), 212–224. 10.1080/07420528.2016.1260026 27996307

[hbm24945-bib-0003] Carter, M. D. , & Juurlink, D. N. (2012). Melatonin. CMAJ, 184(17), 1923. doi: 10.1503/cmaj.111765 22508974 PMC3503905

[hbm24945-bib-0004] Chao‐Gan, Y. , & Yu‐Feng, Z. (2010). DPARSF: A MATLAB toolbox for “pipeline” data analysis of resting‐state fMRI. Frontiers in Systems Neuroscience, 4, 13 10.3389/fnsys.2010.00013 20577591 PMC2889691

[hbm24945-bib-0005] Chee, M. W. , & Choo, W. C. (2004). Functional imaging of working memory after 24 hr of total sleep deprivation. The Journal of Neuroscience, 24(19), 4560–4567. 10.1523/jneurosci.0007-04.2004 15140927 PMC6729385

[hbm24945-bib-0006] Chee, M. W. , Chuah, L. Y. , Venkatraman, V. , Chan, W. Y. , Philip, P. , & Dinges, D. F. (2006). Functional imaging of working memory following normal sleep and after 24 and 35 h of sleep deprivation: Correlations of fronto‐parietal activation with performance. NeuroImage, 31(1), 419–428. 10.1016/j.neuroimage.2005.12.001 16427321

[hbm24945-bib-0007] Cho, K. (2001). Chronic “jet lag” produces temporal lobe atrophy and spatial cognitive deficits. Nature Neuroscience, 4(6), 567–568. 10.1038/88384 11369936

[hbm24945-bib-0008] Coutinho, J. F. , Goncalves, O. F. , Maia, L. , Fernandes Vasconcelos, C. , Perrone‐McGovern, K. , Simon‐Dack, S. , … Sampaio, A. (2015). Differential activation of the default mode network in jet lagged individuals. Chronobiology International, 32(1), 143–149. 10.3109/07420528.2014.955187 25180985

[hbm24945-bib-0009] Crawford, J. R. , & Henry, J. D. (2004). The positive and negative affect schedule (PANAS): Construct validity, measurement properties and normative data in a large non‐clinical sample. The British Journal of Clinical Psychology, 43(Pt 3), 245–265. 10.1348/0144665031752934 15333231

[hbm24945-bib-0010] Drust, B. , Waterhouse, J. , Atkinson, G. , Edwards, B. , & Reilly, T. (2005). Circadian rhythms in sports performance—an update. Chronobiology International, 22(1), 21–44. 10.1081/CBI-200041039 15865319

[hbm24945-bib-0011] Fan, L. , Li, H. , Zhuo, J. , Zhang, Y. , Wang, J. , Chen, L. , … Jiang, T. (2016). The human Brainnetome atlas: A new brain atlas based on connectional architecture. Cerebral Cortex, 26(8), 3508–3526. 10.1093/cercor/bhw157 27230218 PMC4961028

[hbm24945-bib-0012] Gander, P. H. , Nguyen, D. , Rosekind, M. R. , & Connell, L. J. (1993). Age, circadian rhythms, and sleep loss in flight crews. Aviation, Space, and Environmental Medicine, 64(3), 189–195. 8447798

[hbm24945-bib-0013] Gorfine, T. , & Zisapel, N. (2009). Late evening brain activation patterns and their relation to the internal biological time, melatonin, and homeostatic sleep debt. Human Brain Mapping, 30(2), 541–552. 10.1002/hbm.20525 18095278 PMC6871121

[hbm24945-bib-0014] Herxheimer, A. (2008). Jet lag. BMJ Clinical Evidence, 2008, 2303. PMC2907932 19445780

[hbm24945-bib-0015] Herxheimer, A. (2014). Jet lag. BMJ Clinical Evidence, 2014, 2303. PMC4006102 24780537

[hbm24945-bib-0016] Herxheimer, A. , & Petrie, K. J. (2002). Melatonin for the prevention and treatment of jet lag. Cochrane Database System Review(2), Cd001520. 10.1002/14651858.cd001520 12076414

[hbm24945-bib-0017] Hirschfeld, U. , Moreno‐Reyes, R. , Akseki, E. , L'Hermite‐Baleriaux, M. , Leproult, R. , Copinschi, G. , & Van Cauter, E. (1996). Progressive elevation of plasma thyrotropin during adaptation to simulated jet lag: Effects of treatment with bright light or zolpidem. The Journal of Clinical Endocrinology and Metabolism, 81(9), 3270–3277. 10.1210/jcem.81.9.8784082 8784082

[hbm24945-bib-0018] Hogervorst, E. , Huppert, F. , Matthews, F. E. , & Brayne, C. (2008). Thyroid function and cognitive decline in the MRC cognitive function and ageing study. Psychoneuroendocrinology, 33(7), 1013–1022. 10.1016/j.psyneuen.2008.05.008 18640783

[hbm24945-bib-0019] Iggena, D. , Winter, Y. , & Steiner, B. (2017). Melatonin restores hippocampal neural precursor cell proliferation and prevents cognitive deficits induced by jet lag simulation in adult mice. Journal of Pineal Research, 62(4): e12397. 10.1111/jpi.12397 28178375

[hbm24945-bib-0020] Karten, A. , Pantazatos, S. P. , Khalil, D. , Zhang, X. , & Hirsch, J. (2013). Dynamic coupling between the lateral occipital‐cortex, default‐mode, and frontoparietal networks during bistable perception. Brain Connectivity, 3(3), 286–293. 10.1089/brain.2012.0119 23510237 PMC3685318

[hbm24945-bib-0021] Latora, V. , & Marchiori, M. (2001). Efficient behavior of small‐world networks. Physical Review Letters, 87(19), 198701 10.1103/PhysRevLett.87.198701 11690461

[hbm24945-bib-0022] Morgenthaler, T. I. , Lee‐Chiong, T. , Alessi, C. , Friedman, L. , Aurora, R. N. , Boehlecke, B. , … Zak, R. (2007). Practice parameters for the clinical evaluation and treatment of circadian rhythm sleep disorders. An American Academy of sleep medicine report. Sleep, 30(11), 1445–1459. 10.1093/sleep/30.11.1445 18041479 PMC2082098

[hbm24945-bib-0023] Muto, V. , Jaspar, M. , Meyer, C. , Kusse, C. , Chellappa, S. L. , Degueldre, C. , … Maquet, P. (2016). Local modulation of human brain responses by circadian rhythmicity and sleep debt. Science, 353(6300), 687–690. 10.1126/science.aad2993 27516598

[hbm24945-bib-0024] Muto, V. , Shaffii‐le Bourdiec, A. , Matarazzo, L. , Foret, A. , Mascetti, L. , Jaspar, M. , … Maquet, P. (2012). Influence of acute sleep loss on the neural correlates of alerting, orientating and executive attention components. Journal of Sleep Research, 21(6), 648–658. 10.1111/j.1365-2869.2012.01020.x 22594455

[hbm24945-bib-0025] Niu, R. , Lei, D. , Chen, F. , Chen, Y. , Suo, X. , Li, L. , … Gong, Q. (2018). Disrupted grey matter network morphology in pediatric posttraumatic stress disorder. NeuroImage: Clinical, 18, 943–951. 10.1016/j.nicl.2018.03.030 29876279 PMC5988464

[hbm24945-bib-0026] Plante, D. T. , Trksak, G. H. , Jensen, J. E. , Penetar, D. M. , Ravichandran, C. , Riedner, B. A. , … Harper, D. G. (2014). Gray matter‐specific changes in brain bioenergetics after acute sleep deprivation: A 31P magnetic resonance spectroscopy study at 4 tesla. Sleep, 37(12), 1919–1927. 10.5665/sleep.4242 25325507 PMC4548516

[hbm24945-bib-0027] Postuma, R. B. , & Dagher, A. (2006). Basal ganglia functional connectivity based on a meta‐analysis of 126 positron emission tomography and functional magnetic resonance imaging publications. Cerebral Cortex, 16(10), 1508–1521. 10.1093/cercor/bhj088 16373457

[hbm24945-bib-0028] Power, J. D. , Barnes, K. A. , Snyder, A. Z. , Schlaggar, B. L. , & Petersen, S. E. (2012). Spurious but systematic correlations in functional connectivity MRI networks arise from subject motion. NeuroImage, 59(3), 2142–2154. 10.1016/j.neuroimage.2011.10.018 22019881 PMC3254728

[hbm24945-bib-0029] Ramanaiah, N. V. , Franzen, M. , & Schill, T. (1983). A psychometric study of the state‐trait anxiety inventory. Journal of Personality Assessment, 47(5), 531–535. 10.1207/s15327752jpa4705_14 6644527

[hbm24945-bib-0030] Reilly, T. , Atkinson, G. , & Budgett, R. (2001). Effect of low‐dose temazepam on physiological variables and performance tests following a westerly flight across five time zones. International Journal of Sports Medicine, 22(3), 166–174. 10.1055/s-2001-16379 11354518

[hbm24945-bib-0031] Reilly, T. , Waterhouse, J. , & Edwards, B. (2008). A review on some of the problems associated with long‐distance journeys. La Clinica Terapeutica, 159(2), 117–127. 18463771

[hbm24945-bib-0033] Rubinov, M. , & Sporns, O. (2010). Complex network measures of brain connectivity: Uses and interpretations. NeuroImage, 52(3), 1059–1069. 10.1016/j.neuroimage.2009.10.003 19819337

[hbm24945-bib-0034] Sack, R. L. (2010). Clinical practice. Jet lag. The New England Journal of Medicine, 362(5), 440–447. 10.1056/NEJMcp0909838 20130253

[hbm24945-bib-0035] Shao, Y. , Wang, L. , Ye, E. , Jin, X. , Ni, W. , Yang, Y. , … Yang, Z. (2013). Decreased thalamocortical functional connectivity after 36 hours of total sleep deprivation: Evidence from resting state FMRI. PLoS One, 8(10), e78830 10.1371/journal.pone.0078830 24205327 PMC3808277

[hbm24945-bib-0032] Spitzer, R. L. , Terman, M. , Williams, J. B. W. , Terman, J. S. , Malt, U. F. , Singer, F. , & Lewy, A. J. (1999). Jet lag: Clinical features, validation of a new syndrome‐specific scale, and lack of response to melatonin in a randomized, double‐blind trial. American Journal of Psychiatry, 156(9), 1392–1396. 10.1176/ajp.156.9.1392 10484950

[hbm24945-bib-0036] Sporns, O. , Tononi, G. , & Kotter, R. (2005). The human connectome: A structural description of the human brain. PLoS Computational Biology, 1(4), e42 10.1371/journal.pcbi.0010042 16201007 PMC1239902

[hbm24945-bib-0037] Sterpenich, V. , Albouy, G. , Boly, M. , Vandewalle, G. , Darsaud, A. , Balteau, E. , … Maquet, P. (2007). Sleep‐related hippocampo‐cortical interplay during emotional memory recollection. PLoS Biology, 5(11), e282 10.1371/journal.pbio.0050282 17958471 PMC2039770

[hbm24945-bib-0038] Thomas, M. , Sing, H. , Belenky, G. , Holcomb, H. , Mayberg, H. , Dannals, R. , … Redmond, D. (2000). Neural basis of alertness and cognitive performance impairments during sleepiness. I. Effects of 24 h of sleep deprivation on waking human regional brain activity. Journal of Sleep Research, 9(4), 335–352. 10.1046/j.1365-2869.2000.00225.x 11123521

[hbm24945-bib-0039] Wahlin, A. , Bunce, D. , & Wahlin, T. B. (2005). Longitudinal evidence of the impact of normal thyroid stimulating hormone variations on cognitive functioning in very old age. Psychoneuroendocrinology, 30(7), 625–637. 10.1016/j.psyneuen.2005.01.010 15854779

[hbm24945-bib-0040] Waterhouse, J. , Reilly, T. , & Atkinson, G. (1997). Jet‐lag. The Lancet, 350(9091), 1611–1616. 10.1016/s0140-6736(97)07569-7 9393352

[hbm24945-bib-0041] Watts, D. J. , & Strogatz, S. H. (1998). Collective dynamics of “small‐world” networks. Nature, 393(6684), 440–442. 10.1038/30918 9623998

[hbm24945-bib-0042] Wright, J. E. , Vogel, J. A. , Sampson, J. B. , Knapik, J. J. , Patton, J. F. , & Daniels, W. L. (1983). Effects of travel across time zones (jet‐lag) on exercise capacity and performance. Aviation, Space, and Environmental Medicine, 54(2), 132–137. 6838449

[hbm24945-bib-0043] Zhang, J. , Wang, J. , Wu, Q. , Kuang, W. , Huang, X. , He, Y. , & Gong, Q. (2011). Disrupted brain connectivity networks in drug‐naive, first‐episode major depressive disorder. Biological Psychiatry, 70(4), 334–342. 10.1016/j.biopsych.2011.05.018 21791259

[hbm24945-bib-0044] Zhao, Y. , Li, J. , Liu, X. , Song, Y. , Wang, R. , Yang, Z. , & Liu, J. (2016). Altered spontaneous neural activity in the occipital face area reflects behavioral deficits in developmental prosopagnosia. Neuropsychologia, 89, 344–355. 10.1016/j.neuropsychologia.2016.05.027 27475965

[hbm24945-bib-0045] Zhu, Y. , Feng, Z. , Xu, J. , Fu, C. , Sun, J. , Yang, X. , … Qin, W. (2016). Increased interhemispheric resting‐state functional connectivity after sleep deprivation: A resting‐state fMRI study. Brain Imaging and Behavior, 10(3), 911–919. 10.1007/s11682-015-9490-5 26634366

[hbm24945-bib-0046] Zisapel, N. (2018). New perspectives on the role of melatonin in human sleep, circadian rhythms and their regulation. British Journal of Pharmacology, 175, 3190–3199. 10.1111/bph.14116 29318587 PMC6057895

