# Supplementary figures and images for: The effect of jet lag on the human brain: A neuroimaging study
Source: Hum Brain Mapp. 2020 Mar 3;41(9):2281–91. doi: 10.1002/hbm.24945 (PMC7268074; doi:10.1002/hbm.24945)

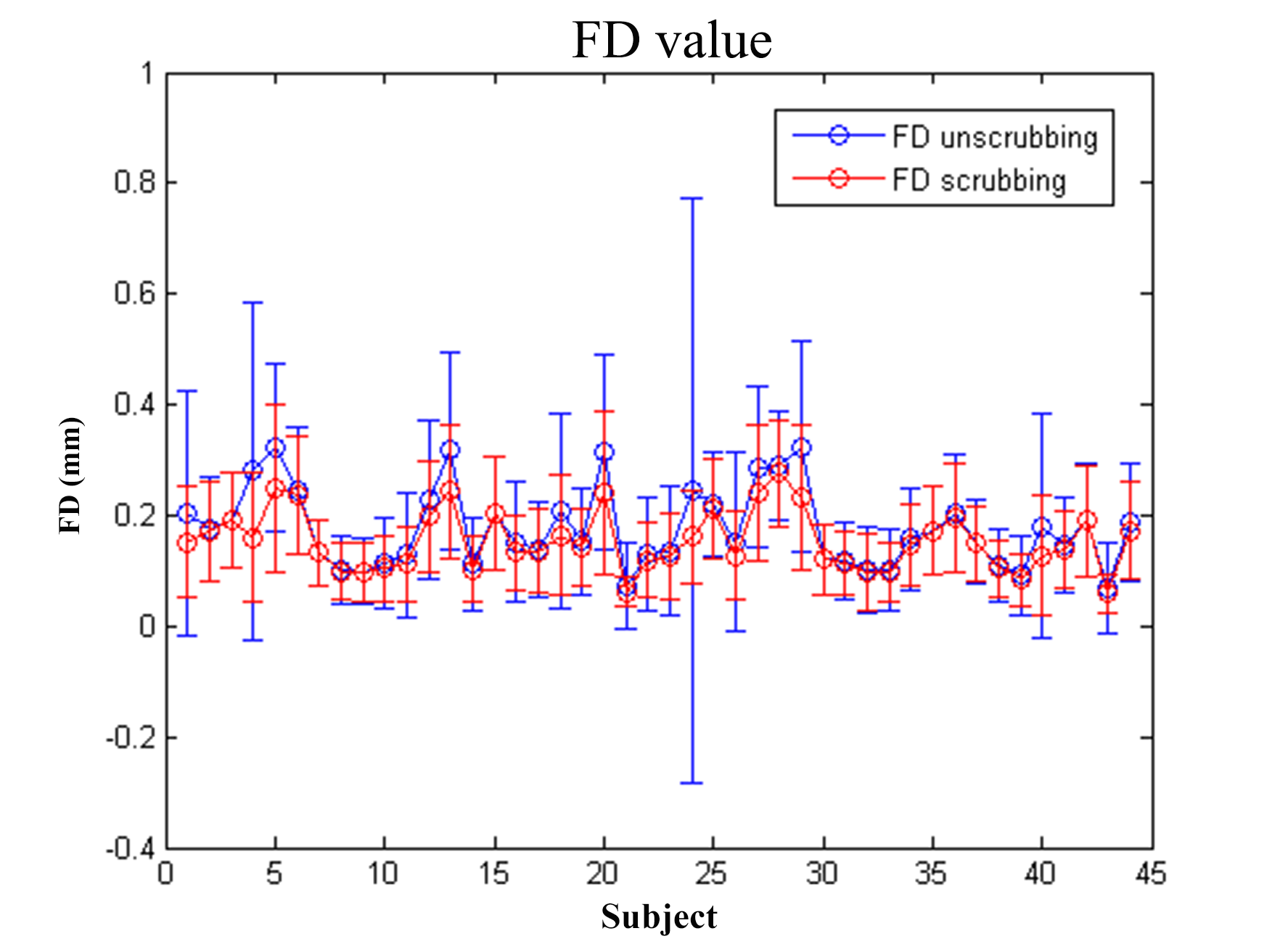

Supplement: Supplementary file 1 — Supplementary Figure S1 The figure below shows mean FD across the entire time series for each subject. The blue line represents FD values before scrubbing, while the red line means the FD values after scrubbing. FD, frame‐wise displacement. [file HBM-41-2281-s001.tif]
